# Sequence Analysis of *Macaca mulatta TRIM4* and Its Role in the Interferon Pathway

**DOI:** 10.3389/fvets.2022.805301

**Published:** 2022-02-15

**Authors:** Mengmeng Zhao, Huawei Li, Hang Zhang, Huiyang Sha, Liangzong Huang, Ruining Wang

**Affiliations:** ^1^Department of Veterinary Medicine, School of Life Science and Engineering, Foshan University, Foshan, China; ^2^Henan University of Animal Husbandry and Economy, Zhengzhou, China

**Keywords:** TRIM4, interferon, innate immunity, *Macaca mulatta*, *CCL5*

## Abstract

Monkey diseases are becoming increasingly severe, and some may be transmitted to humans through direct and indirect contact. Innate immunity is the first line of defense against foreign microorganisms. It is of great significance to explore the immune characteristics of monkey and human diseases. TRIM4, an important immune molecule in *Macaca mulatta*, was cloned and its immunological characteristics were preliminarily explored. The results showed that *Macaca mulatta TRIM4* was in the same branch as human *TRIM4*. Overexpression of TRIM4 increased the mRNA levels of interferon (IFN)-*alpha, IFN-beta, RIG-I, MAVS, IRF3, IRF7, OAS1, IFIT3, and CCL5*, TRIM4 up-regulated the activities of IFN-beta, NF-κB, and ISRE reporter. In contrast, inhibiting *TRIM4* expression by small interfering RNA (siRNA) down-regulated the IFN pathway. In summary, *Macaca mulatta TRIM4* plays an essential role in the IFN pathway.

## Introduction

Innate immunity plays an important role in the resistance to foreign pathogen invasion. Foreign microorganisms are recognized by pattern recognition receptors (PRRs), including toll-like receptors (TLRs), NOD-like receptors (NLRs), RIG-I-like receptors (RLRs), and C-type lectin receptors (CLRs) (1). The body stimulates a series of signaling proteins and pathways and activates multiple cytokines, which further stimulate downstream signaling molecules that act as antiviral molecules and play an essential role in eliminating invading pathogens (2–5).

Tripartite motif protein (TRIM) is a vital signaling molecule that plays an essential role in many cellular processes, including cell proliferation, differentiation, carcinogenesis, innate immunity, and apoptosis (6). For example, TRIM79α inhibits the replication of Tick-borne encephalitis virus by degrading RNA polymerase (7). TRIM28 inhibits the infection of murine leukemia virus and promotes virus entry into the latent period (8). TRIM52 interacts with the NS2A protein of the Japanese encephalitis virus and degrades the NS2A protein. TRIM41 ubiquitinates and degrades nucleoprotein and inhibits influenza virus replication by interacting with the influenza virus nucleoprotein (9). TRIM11 has been shown to inhibit the invasion, replication, and release of human immunodeficiency virus 1(HIV-1), plays an antiviral role, carries out ubiquitination modification of the protein, and negatively regulates interferon regulatory factor 3(IRF3) and activates and promotes the infection of HSV-1 (10).

Studies have shown that human TRIM4, an essential regulatory molecule of RIG-I, participates in the interferon (IFN) pathway (11). However, TRIM4 has not yet been reported in monkeys. In this study, the *TRIM4* gene was cloned from *rhesus macaque (Macaca mulata)*, and the relationship between TRIM4 and IFN was preliminarily explored, providing a theoretical basis for treating diseases.

## Materials and Methods

### Cells

MARC145 cells were cultured in Dulbecco's Modified Eagle's Medium (DMEM) (Solarbio, Beijing, China, #31600-034) together with 10% fetal bovine serum (Sijiqing, ZhejiangTianhang Biotechnology Co. Ltd., China) at 37°C in a humidified atmosphere containing 5% CO_2_. There was no mycoplasma contamination in the MARC145 cells.

### TRIM4 Cloning

*Macaca mulatta* was provided by Guangzhou Xusheng Biotechnology Co. Ltd. The ethics committee of Foshan University has approved all animal experiments. *Macaca mulatta* liver samples were used for RNA extraction with TRIzol reagent (Beyotime, Shanghai, China, #R0016). HiScript III 1st Strand cDNA Synthesis Kit (+gDNA wiper) (Vazyme, Nanjing, China, #R312) was used for reverse transcription. Finally, complementary DNA (cDNA) was used for subsequent molecular cloning experiments.

TRIM4 cloning was performed using a standard procedure for molecular cloning. After double digestion with *Xho* I and *Bam*H I, PCR product was ligated to pCMV-3xFLAG-7.1. The primers of TRIM4 were shown in Supplementary Table 1. The *TRIM4* upstream and downstream primers were designed according to the CDS region of the *TRIM4* sequence, the *Xho* I restriction site was incorporated in the upstream primer, the downstream primer contains a *Bam*H I restriction site. The 2× phanta master mix (Vazyme, Nanjing, China, #DC401) was used for the PCR amplification, and reaction conditions were 95°C for 5 min, followed by 40 cycles at 95°C for 30 s, 53°C for 30 s and 72°C for 120 s. The Mix & Go! *E. coli* Transformation Kit (Zymo Research, Irvine, CA) was used for transformation. PurePlasmid Mini Kit (CoWin Biosciences, Beijing, China) was used for plasmid extraction.

### Phylogenetic Tree Construction

The phylogenetic tree was constructed using the neighbor-joining method with MEGA 5.0 (12).

### Transfection

The plasmids, poly(I:C) (InvivoGen, San Diego, CA, #tlrl-pic) and poly(dA:dT) (InvivoGen, #tlrl-patn) were transfected using Lipofectamine 3000 Transfection Reagent (Invitrogen, #L3000001).

*Macaca mulatta TRIM4* siRNA (si-TRIM4) and non-targeting control siRNA (si-NC) were transfected into MARC145 cells using RNAi-mate (GenePharma Co., Ltd., Suzhou, China, #G04001). The si-TRIM4 and si-NC were synthesized by GenePharma Co., Ltd. Knockdown efficiency was verified by western blotting.

FLAG-tagged TRIM4 (FlAG-TRIM4) plasmid (300 ng) or si-TRIM4 (60 nM), reporter plasmid (200 ng), and RL-TK (20 ng) (Promega, Madison, WI) were transfected into MARC145 cells using Lipofectamine 3000. After 48 h, reporter activity was measured by a luciferase reporter assay (Promega, Madison, WI, #E1910). The IFN-beta, nuclear factor-κB (NF-κB), and ISRE reporter plasmid are bought from Beyotime (Shanghai, China) and kept in the laboratory as previously described (13).

### Real-Time PCR

The mRNA expression levels of *IFN-alpha, IFN-beta, retinoic-acid–inducible gene I (RIG-I), mitochondria antiviral signaling protein* (MAVS)*, interferon regulatory factor (IRF) 3, IRF7, 2*′*-5*′ *Oligoadenylate synthetases (OAS)1, interferon-induced protein with tetratricopeptide repeats (IFIT3), and CCL5* were determined by real-time PCR on a 7500 Fast Real-time PCR system (Applied Biosystems, Foster City, CA). The PCR procedure and quantification were performed as described in a previous study (13). Total RNA was extracted using TRIzol (Beyotime, Shanghai, China, #R0016), and then 500 ng RNA was reverse transcribed by Hiscript III 1st strand cDNA synthesis kit (+gDNA wiper) (Vazyme, Nanjing, China, #R312), the cDNA was stored for real-time PCR. The volume of real-time PCR reaction was 20 μL, in which 10 μL BeyoFast SYBR Green qPCR Mix (Beyotime, Shanghai, China, #D7265), 6 μL ddH_2_O, 2 μL cDNA, 2 μL upstream and downstream primers were included. The real-time PCR conditions were 95°C for 2 min, followed by 40 cycles at 95°C for 15 s and 60°C for 30 s, the primers are shown in Supplementary Table 1. The relative expression of the target gene was calculated by 2^−ΔΔCt^ relative quantitative method.

### Western Blotting

Western blotting was performed as described previously (14–18). Cells were lysed using RIPA Lysis buffer; these cell lysates were centrifuged for 10 min at 13,000 g. The supernatants were collected and mixed with 5x SDS-PAGE sample loading buffer. Then 40 μg proteins were loaded; these proteins were separated by 12% SDS-PAGE separating gel (Beyotime, Shanghai, China, #P0459S) and transferred to Polyvinylidene difluoride (PVDF) membranes. PVDF membranes were blocked with 5% skimmed milk at 25°C for 1 h and incubated with diluted primary antibodies at 4°C for 15 h. After three washes with 1x TBS (each time for 10 min), membranes were incubated with horseradish peroxidase (HRP) conjugated secondary antibodies at 25°C for 1 h, then blots were subjected to detection using Clarity Western ECL Substrate and film in a dark room. First antibodies were anti-FLAG monoclonal antibody (Bioss Antibodies, Beijing, China, #bs-0879R), anti-β-actin polyclonal antibody (Solarbio, Beijing, China, #K200058M), anti-TRIM4 polyclonal antibody (ABclonal, Wuhan, China, #A15922), anti-GAPDH antibody (Beyotime, Shanghai, China, #AF0006), anti-IRF3 monoclonal antibody (Santa Cruz Biotechnology, Dallas, TX, #sc-358914), anti-phospho-IRF3 monoclonal antibody (Cell Signaling Technology, Danvers, MA, #37829), IRF7 monoclonal antibody (Cell Signaling Technology, #72073), anti-phospho-IRF7 monoclonal antibody (Cell Signaling Technology, #24129), anti-NF-κB p65 (Bioss Antibodies, bs-0465R), anti-phospho-NF-κB p65 (Ser536) (Cell Signaling Technology, #3033S), Second antibodies were horseradish peroxide-conjugated rabbit anti-mouse IgG antibody (Abclonal, Wuhan, China, #WH166568) and mouse anti-rabbit IgG antibody (Santa Cruz Biotechnology, #sc-2357).

### Infections

MARC145 cells were infected with the PRRSV-2 BJ-4 strain (GenBank accession no. AF331831) for 1 h. Then, the medium was changed to DMEM with 2% FBS, and samples were obtained at the indicated time points.

### Statistical Analysis

There are three biological repeats in all the experiments. Mean ± standard deviation is the method of displaying data, and paired student's *t*-test and GraphPad Prism 5.0 (GraphPad Software, San Diego, CA) were used to analyze the data. Values of *p* < 0.05 were considered statistically significant.

## Results

### Phylogenetic Analysis of TRIM4 From Different Species

First, we cloned the *Macaca mulata TRIM4* gene from healthy *Macaca mulata* liver tissue. *TRIM4* gene is 1,425 bp in length and encodes 475 amino acids. Phylogenetic tree analysis showed that *Macaca mulata TRIM4* is in the same branch as humans and pan troglodytes, and they share a close genetic relationship (Supplementary Figure 1).

### Overexpression of TRIM4 Activates the IFN Pathway

**Figure 1:**
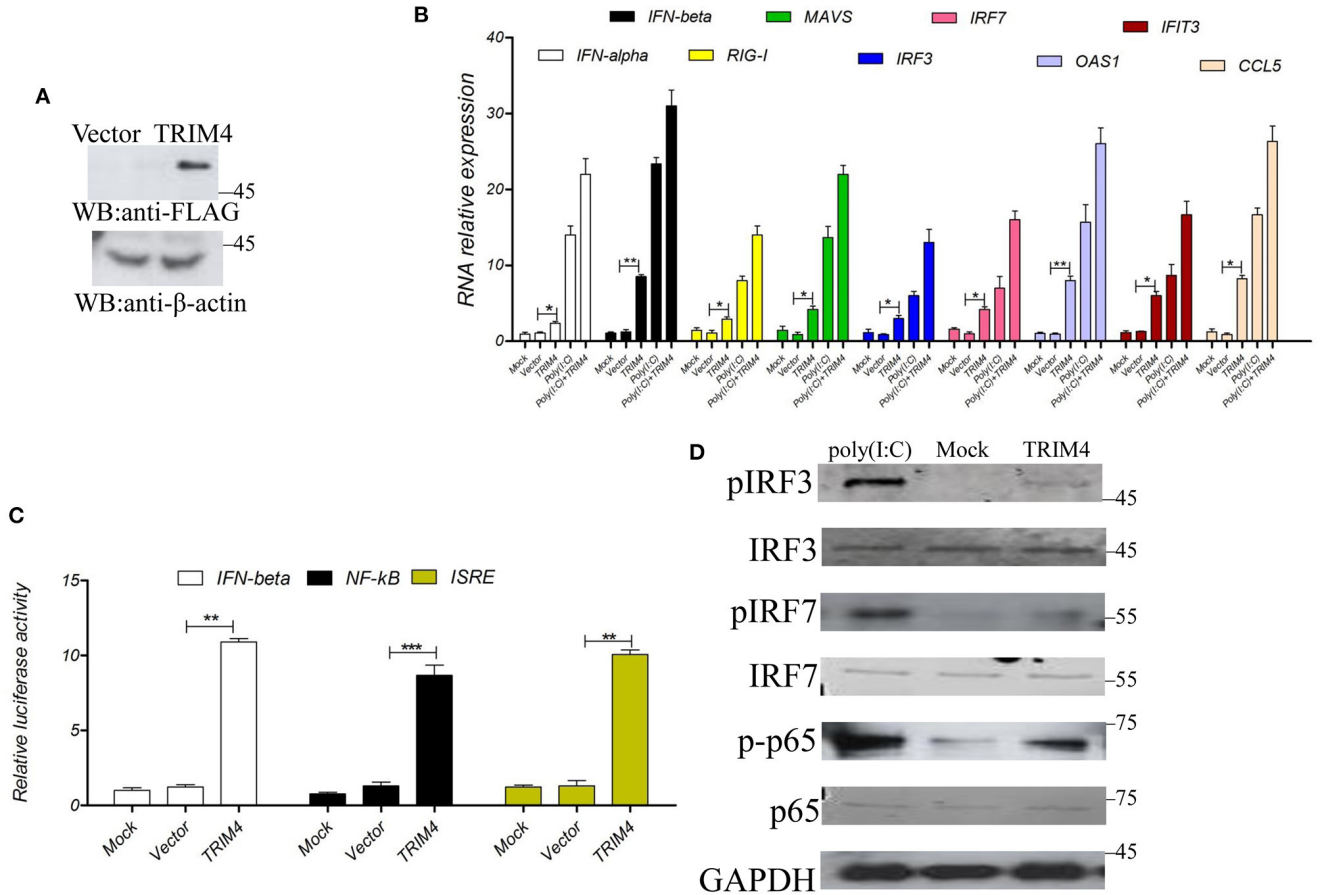
*Macaca mulata* TRIM4 overexpression activates the interferon (IFN) pathway. **(A)** The TRIM4 plasmid was transfected into MARC145 cells. After 48 h, western blotting was performed with the indicated antibody. **(B)** After TRIM4 transfection in MARC145 cells, RNA was extracted 24 h post-transfection. At the same time, a poly(I:C) (1.0 μg/mL) control group was set up, 1.0 μg/mL poly(I:C) treated MARC145 cells, cellular RNA was extracted at 9 h post-transfection. The mRNA levels of *IFN-alpha, IFN-beta, RIG-I, MAVS, IRF3, IRF7, OAS1, IFIT3, and CCL5* were determined by qPCR. **(C)** Overexpression of *Macaca mulata* TRIM4 promoted the activities of IFN-beta, NF-κB, and ISRE promoters. The TRIM4 plasmid (300 ng), 200 ng of IFN-beta reporter plasmid, NF-κB reporter plasmid, ISRE reporter plasmid, and 50 ng of ng RL-TK plasmid were co-transfected into MARC145 cells in a 24-well plate, and the activity of these reporters was detected at 48 h post-transfection. **(D)** Overexpression of TRIM4 increases the phosphorylated levels of interferon-related molecules. The TRIM4 plasmid (500 ng) were transfected into MARC145 cells in a 24-well plate, after 24 h post-transfection, 1.0 μg/mL poly(I:C) was transfected, cell lysates were blotted with the indicated antibodies. ^*^P < 0.05; ^**^P < 0.01; ^***^P < 0.001.

To study the immunological characteristics of TRIM4, we first explored the role of TRIM4 in the IFN pathway. TRIM4 plasmid was transfected into MARC145 cells, after 48 h, the mRNA expression of *IFN-alpha, IFN-beta, RIG-I, MAVS, IRF3, IRF7, OAS1, IFIT3, and CCL5* was determined by qPCR, and the poly(I:C) control group was set up. First, TRIM4 was well expressed in cells (Figure 1A), then the results indicated that poly(I:C) and TRIM4 increased the mRNA levels of *IFN-alpha, IFN-beta, RIG-I, MAVS, IRF3, IRF7, OAS1, IFIT3, and CCL5* (Figure 1B). Furthermore, we co-transfected TRIM4 and promoter plasmids (IFN-beta, NF-κB, and ISRE) into MARC145 cells and measured their promoter activity at 48 h post-transfection. The results indicated that TRIM4 increased the activity of these promoters (Figure 1C). Western blotting results showed that poly(I:C) and TRIM4 significantly increased the levels of phosphorylated IRF3, phosphorylated IRF7 and phosphorylated NF-κB p65 levels (Figure 1D).

### *TRIM4* SiRNA Inhibits the IFN Pathway

**Figure 2:**
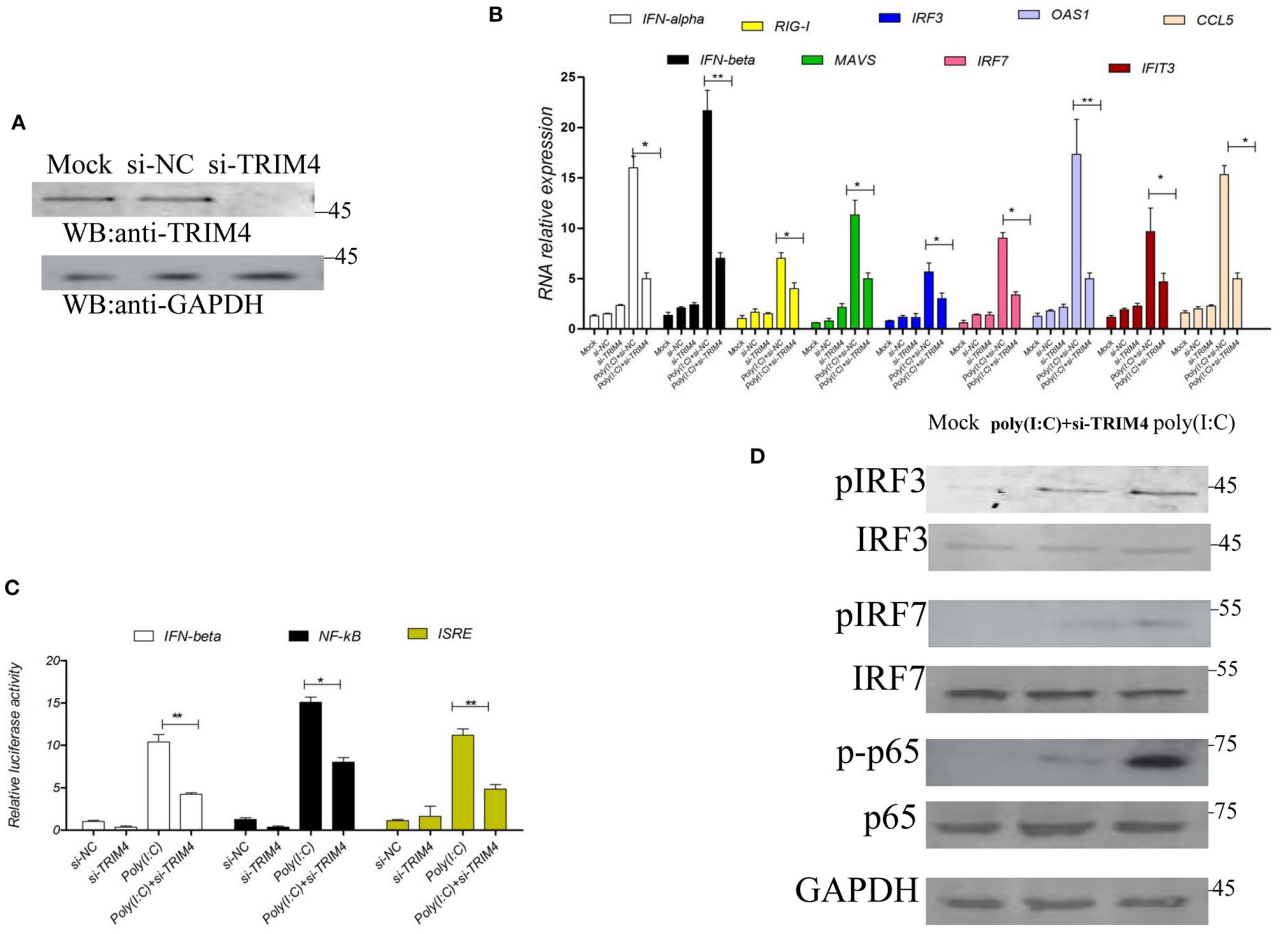
*Macaca mulata TRIM4* siRNA inhibits the IFN pathway. **(A)** The qPCR was used to determine the knockdown efficiency of *TRIM4* siRNA. *TRIM4* siRNA (60 nM) was transfected into MARC145 cells. After 48 h, the expression of *TRIM4* in MARC145 cells was determined by western blotting. **(B)** The effect of *TRIM4* siRNA on *IFN-beta* and *CCL5* mRNA levels. *TRIM4* siRNA (60 nM) was transfected into MARC145 cells, after 24 h, 1.0 μg/mL poly(I:C) was transfected, the changes of *IFN-alpha, IFN-beta, RIG-I, MAVS, IRF3, IRF7, OAS1, IFIT3, and CCL5* mRNA expression in MARC145 cells were determined by qPCR at 9 h post-transfection. **(C)** The effect of *TRIM4* siRNA on the activity of IFN-beta, NF-κB, and ISRE promoters. *TRIM4* siRNA (60 nM), IFN-beta reporter plasmid (200 ng), NF-κB reporter plasmid, ISRE reporter plasmid, and 50 ng of RL-TK plasmid were transfected into MARC145 cells. After 24 h, 1.0 μg/mL poly(I:C) was transfected, and the activity of the reporter was detected by a reporter test at 9 h post-transfection. **(D)** *TRIM4* siRNA downregulates the phosphorylated levels of interferon-related molecules. The si-TRIM4 (60 nM) were transfected into MARC145 cells in a 24-well plate, after 24 h post-transfection, 1.0 μg/mL poly(I:C) was transfected, cell lysates were blotted with the indicated antibodies. ^*^P < 0.05; ^**^P < 0.01.

Next, we used siRNA to verify the effect of TRIM4 in the IFN pathway; *TRIM4* siRNA was transfected into MARC145 cells, and its knockdown effect on *TRIM4* expression levels was confirmed by western blot. The results indicated that *TRIM4* siRNA effectively knocked down *TRIM4* expression (Figure 2A). Then, we stimulated the MARC145 cells with poly(I:C) for 9 h, and measured the mRNA levels of *IFN-alpha, IFN-beta, RIG-I, MAVS, IRF3, IRF7, OAS1, IFIT3, and CCL5* by qPCR. The results indicated that the mRNA levels of *IFN-alpha, IFN-beta, RIG-I, MAVS, IRF3, IRF7, OAS1, IFIT3, and CCL5* were significantly decreased after *TRIM4* siRNA transfection (Figure 2B). On the other hand, different promoter plasmids and *TRIM4* siRNA were co-transfected into MARC145 cells; after 24 h, the cells were stimulated with poly(I:C) for 9 h, and the promoter activities were tested. The results showed that the activities of IFN-beta, NF-κB, and ISRE promoters decreased significantly after transfection with *TRIM4* siRNA (Figure 2C). Western blotting results showed that si-TRIM4 significantly reduced the protein levels of phosphorylated IRF3, phosphorylated IRF7 and phosphorylated NF-κB p65 (Figure 2D).

### Effect of Different Stimuli on TRIM4

To investigate the effect of porcine reproductive and respiratory syndrome virus (PRRSV) infection on *TRIM4* expression, we infected MARC145 cells with PRRSV at different multiplicities of infection (MOI). The results showed that 0.01, 0.1, and 1 MOI PRRSV infection increased the *Macaca mulata TRIM4* mRNA levels and protein levels (Supplementary Figure 2A). The changes in TRIM4 at different time points of viral infection were further analyzed. The results showed that the expression of TRIM4 was the highest at 36 h post-infection, which was increased by approximately 7-fold, western blotting results showed that the protein level of TRIM4 increased (Supplementary Figure 2B). Further experimental results show that over-expression of TRIM4 inhibit PRRSV replication (Supplementary Figure 2C).

**Figure 3:**
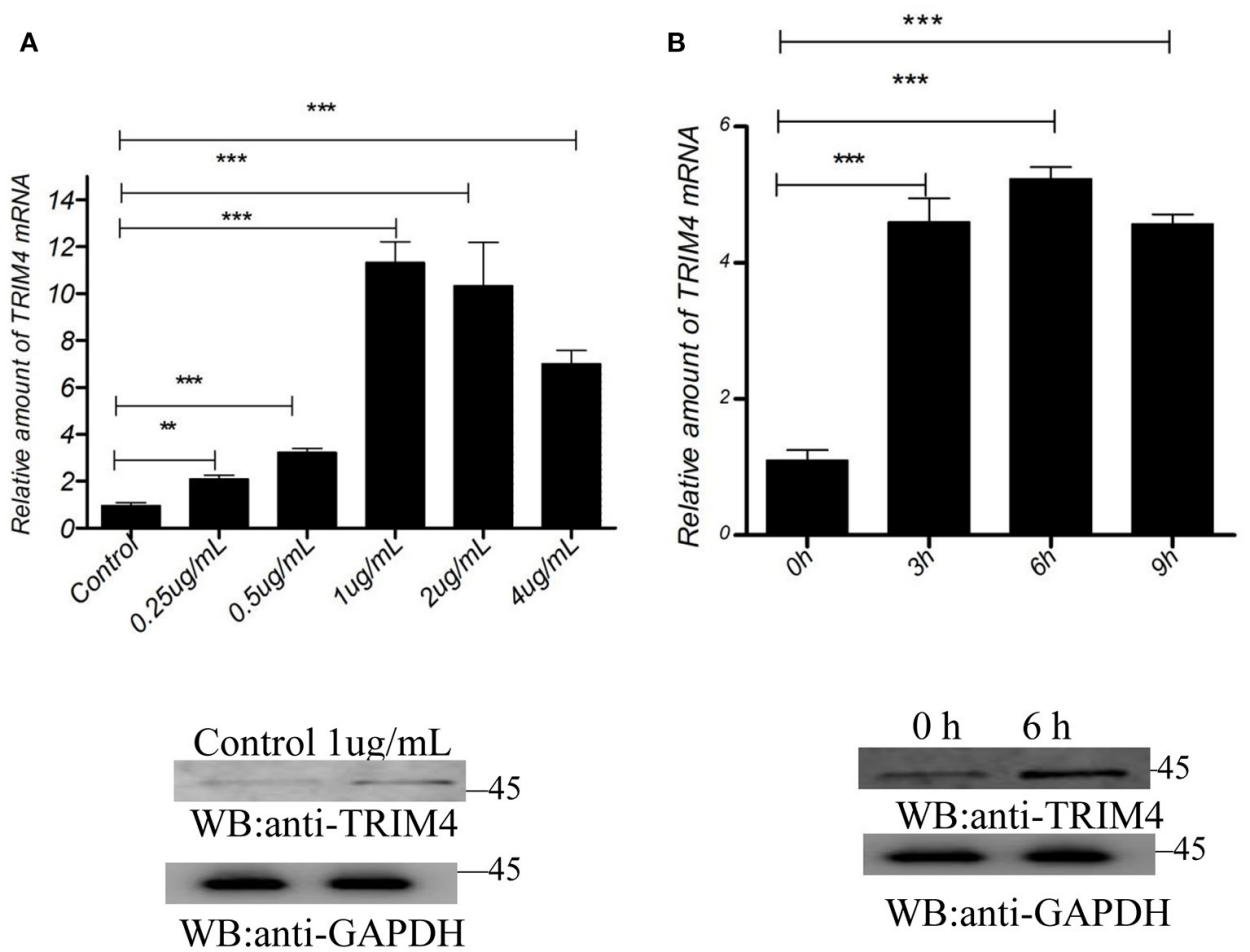
Poly(I:C) response to *TRIM4* expression. **(A)** Effect of different poly(I:C) concentrations on *TRIM4* expression. MARC145 cells were transfected with poly(I:C) at different doses (0, 0.25, 0.5, 1, 2, and 4 μg/mL). After 9 h, the cells were collected, and the changes in *TRIM4* were determined by qPCR and western blotting. **(B)** Evaluation of different time points at the same poly(I:C) concentration on *TRIM4* expression. Poly(I:C) (1.0 μg/mL) was transfected into MARC145 cells. The mRNA level and protein level of *TRIM4* was tested by qPCR and western blotting at the designated time points (0, 3, 6, and 9 h). ^**^P < 0.01; ^***^P < 0.001.

**Figure 4:**
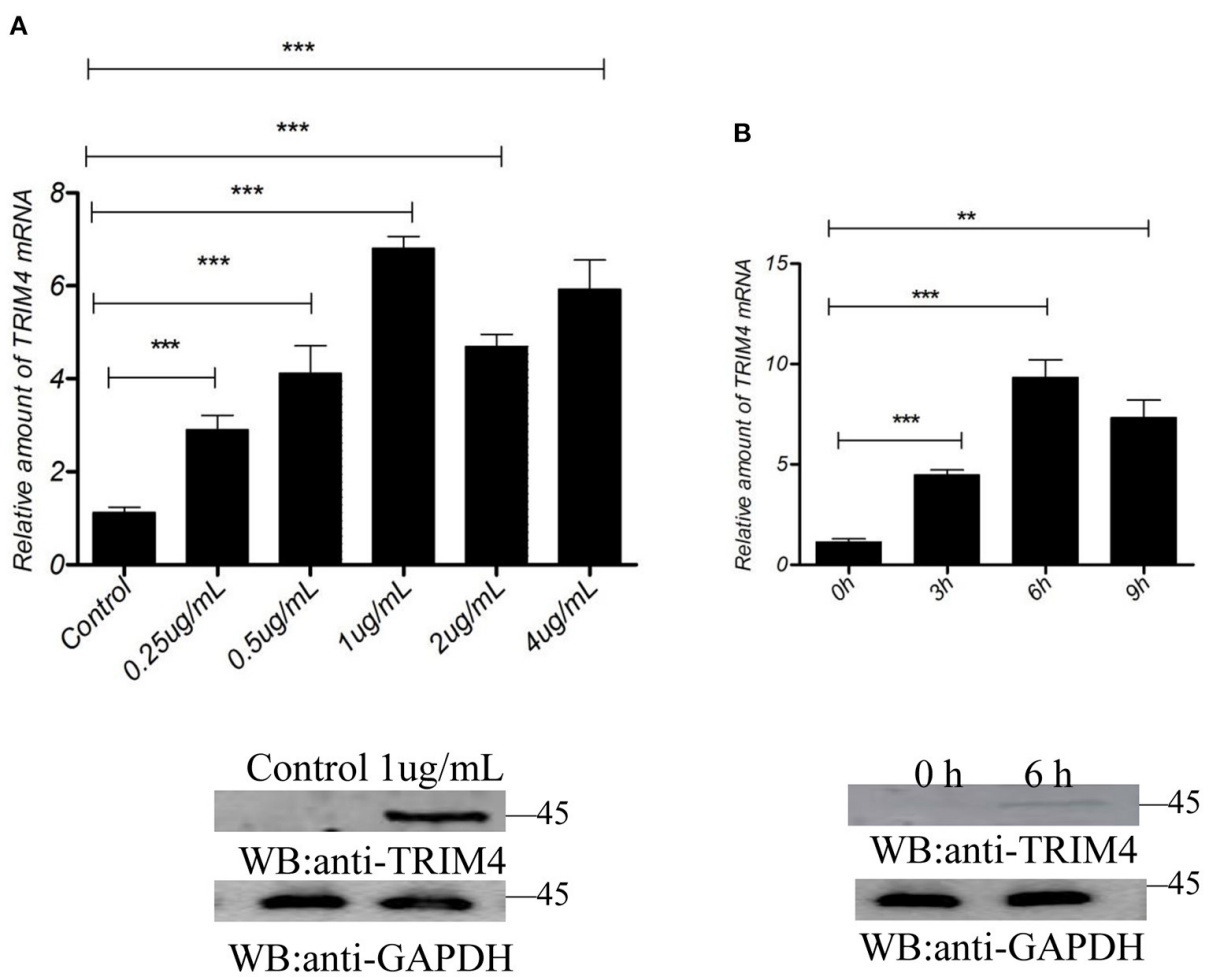
Poly(dA: dT) response to *TRIM4* expression. **(A)** TMARC145 cells were transfected with poly(dA: dT) at different doses (0, 0.25, 0.5, 1, 2, and 4 μg/mL). After 9 h, the changes in *TRIM4* were determined by qPCR and western blotting. **(B)** Evaluation of different time points with the same poly(dA: dT) concentration on *TRIM4* expression. Poly(dA: dT) (1.0 μg/mL) was transfected into MARC145 cells. The expression of *TRIM4* was assessed by qPCR and western blotting at the designated time points (0, 3, 6, and 9 h). ^**^P < 0.01; ^***^P < 0.001.

The results showed that concentrations of 0.25, 0.5, 1, 2, and 4 μg/mL of poly (I:C) increased the expression of *TRIM4* in 9 h (Figure 3A). In addition, time gradient results showed that 1.0 μg/mL poly(dA: dT) increased TRIM4 expression over time (0, 3, 6, and 9 h) (Figure 3B). The results showed that concentrations of 0.25, 0.5, 1, 2, and 4 μg/mL of poly(dA: dT) increased the expression of TRIM4 in 9 h (Figure 4A). In addition, time gradient results showed that 1.0 μg/mL poly(dA: dT) increased TRIM4 expression over time (0, 3, 6, and 9 h) (Figure 4B).

## Discussions

Viral pathogen-associated molecular patterns (PAMPs) are recognized by PRRs, inducing type I IFN and downstream proteins, which play a pivotal role in initiating the antiviral immune response. Type I IFN mediated by PRRs is regulated by post-translational modifications, especially phosphorylation and ubiquitination (2).

TRIM protein is expressed in cellular physiological processes, such as cell differentiation, apoptosis, tumor genesis, and innate immunity (6). An increasing number of studies have shown that TRIM proteins, such as TRIM5 (19), TRIM21 (20), TRIM23 (21), TRIM29 (22), TRIM31 (23), TRIM32 (24), TRIM56 (25), TRIM65 (26), and TRIM26 (27) positively regulate IFNs in immunological effectors of innate immunity against viral infections; whereas TRIM40 (28) negatively regulates the immune response. However, whether *Macaca mulata* TRIM4 regulates immune response is still unknown.

Our study confirmed that TRIM4 is a positive regulator of the type I IFN induction pathway. Overexpression of TRIM4 activates IFN-beta, NF-κB, and ISRE promoters; TRIM4 increases transcription levels of *IFN-alpha, IFN-beta, RIG-I, MAVS, IRF3, IRF7, OAS1, IFIT3, and CCL5*; and *TRIM4* knockdown decreases the activity of IFN-beta, NF-κB, and ISRE promoters, and decreases transcription levels of *IFN-alpha, IFN-beta, RIG-I, MAVS, IRF3, IRF7, OAS1, IFIT3, and CCL5*. These results reveal a critical role of monkey TRIM4 in the immune response against viruses.

The immunological characteristics and nucleic acid information regarding *Macaca mulata TRIM4* were reported first in this study. The above experiments further confirmed that *Macaca mulata TRIM4* is closely genetically related to human TRIM4, and monkeys are a practical animal model to study human diseases. Sequence analysis showed that TRIM4 has the RING, B-Box, and coil-coil domain; these indicate that *Macaca mulata TRIM4* may be functionally similar to human *TRIM4*.

Expression of TRIM4 increases after PRRSV infection in MARC145 cells, indicating that TRIM4 plays a role in the immune regulation of PRRSV. PRRSV activates the expression of TRIM4, prompting an immune response in the host and inhibiting virus replication. The results showed that TRIM4 overexpression inhibits PRRSV replication, indicating that TRIM4 played an antiviral role as a host antiviral factor.

Poly(I:C) is a simulated RNA analog, and after poly(I:C) stimulation, the expression of *TRIM4* increased, which indicated that TRIM4 played a role in the immune regulation of poly(I:C). These results indicate that TRIM4 responses to RNA virus regulation, the poly(I:C) results coincide with PRRSV results.

Poly(dA: dT) is a simulated DNA analog, and after poly(dA: dT) stimulation, the expression of *TRIM4* increased, which indicated that TRIM4 functions in the immune regulation of poly(dA: dT). These results indicate that TRIM4 functions in the regulation of DNA viruses.

Studies on TRIM4 have shown that TRIM4 interacts with transient receptor potential melastatin 8 and regulates its channel function through K423-mediated ubiquitination (29). TRIM4 can interact with RIG-I, which is regulated by K63-linked polyubiquitination and induces CARD tetramers and facilitates RIG-I multimerization and filamentation, thereby activating RIG-I (11). TRIM4 competes with TRIM25 and interacts with RIG-I-CARD. We speculate that *Macaca mulata TRIM4* may contribute to similar regulatory mechanisms and participate in the monkey IFN pathway. *Macaca mulata TRIM4* may play an antiviral role, and these mechanisms will be studied in the future.

In summary, *Macaca mulata TRIM4* is a crucial regulatory molecule in the IFN pathway and functions a vital role in revealing the monkey immune response.

## Conclusions

*Macaca mulata TRIM4* positively regulates IFN-beta in the IFN pathway.

## Data Availability Statement

The original contributions presented in the study are included in the article/Supplementary Material, further inquiries can be directed to the corresponding authors.

## Ethics Statement

The animal study was reviewed and approved by Foshan University.

## Author Contributions

MZ, HL, and HZ performed the experiments and wrote the manuscript. HS analyzed the data. LH and RW conceived and designed the experiments. All authors read and approved the final manuscript.

## Funding

This work received funds from the National Natural Science Foundation of China (31902279, 31902284). The funder had no role in the study design and collection, analysis, and interpretation of the results.

## Conflict of Interest

The authors declare that the research was conducted in the absence of any commercial or financial relationships that could be construed as a potential conflict of interest.

## Publisher's Note

All claims expressed in this article are solely those of the authors and do not necessarily represent those of their affiliated organizations, or those of the publisher, the editors and the reviewers. Any product that may be evaluated in this article, or claim that may be made by its manufacturer, is not guaranteed or endorsed by the publisher.

## Supplementary Material

Supplementary Figure 1 Phylogenetic analysis of TRIM4 from different species. TRIM4 sequences of *Homo sapiens* (NM_033017), *Macaca mulatta* (MZ706948), *Bos taurus* (XM_005225267), *Sus scrofa* (XM_003124333), *Capra hircus* (XM_004021042), *Equus przewalskii* (XM_008536543.1) and *Pan troglodytes* (XM_009453742) were analyzed using Mega 5.0 software, and an evolutionary tree was obtained.

Supplementary Figure 2 Effects of PRRSV infection on *TRIM4* expression and effects of TRIM4 on PRRSV replication. **(A)** Evaluation of different PRRSV MOI on *TRIM4* expression. MARC145 cells were infected with different PRRSV MOIs (0.01, 0.1, and 1), and after 48 h, the changes in *TRIM4* mRNA expression were measured by qPCR and western blotting. **(B)** Evaluation of different time points with the same PRRSV MOI on *TRIM4* expression. MARC145 cells were infected with 1 MOI PRRSV, and changes in *TRIM4* expression were examined by qPCR and western blotting at different time points (0, 12, 24, 36, 48, 60, and 72 h). **(C)** TRIM4 overexpression inhibits PRRSV replication. FLAG-TRIM4 (500 ng) was transfected into Marc-145 cells; cells were inoculated with 1 MOI PRRSV after 24 h, and the supernatant of cells was collected after 48 h post-infection, the virud titer was measured by TCID_50_.

Supplementary Table 1 Primers used in this study.
