# Supplementary figures and images for: Sequence Analysis of Macaca mulatta TRIM4 and Its Role in the Interferon Pathway
Source: Front Vet Sci. 2022 Feb 15;9:805301. doi: 10.3389/fvets.2022.805301 (PMC8887992; doi:10.3389/fvets.2022.805301)

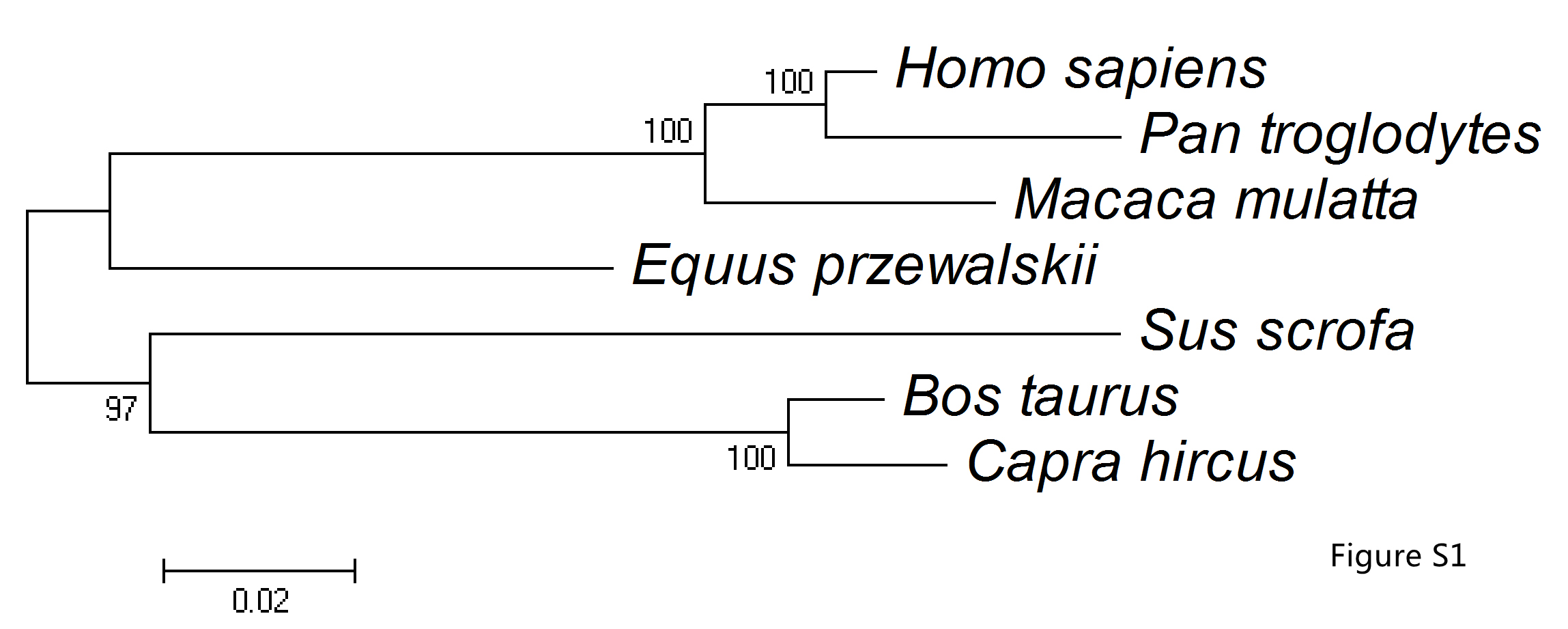

Supplement: Supplementary Figure 1 — Phylogenetic analysis of TRIM4 from different species. TRIM4 sequences of Homo sapiens (NM_033017), Macaca mulatta (MZ706948), Bos taurus (XM_005225267), Sus scrofa (XM_003124333), Capra hircus (XM_004021042), Equus przewalskii (XM_008536543.1) and Pan troglodytes (XM_009453742) were analyzed using Mega 5.0 software, and an evolutionary tree was obtained. [file Image_1.jpg]

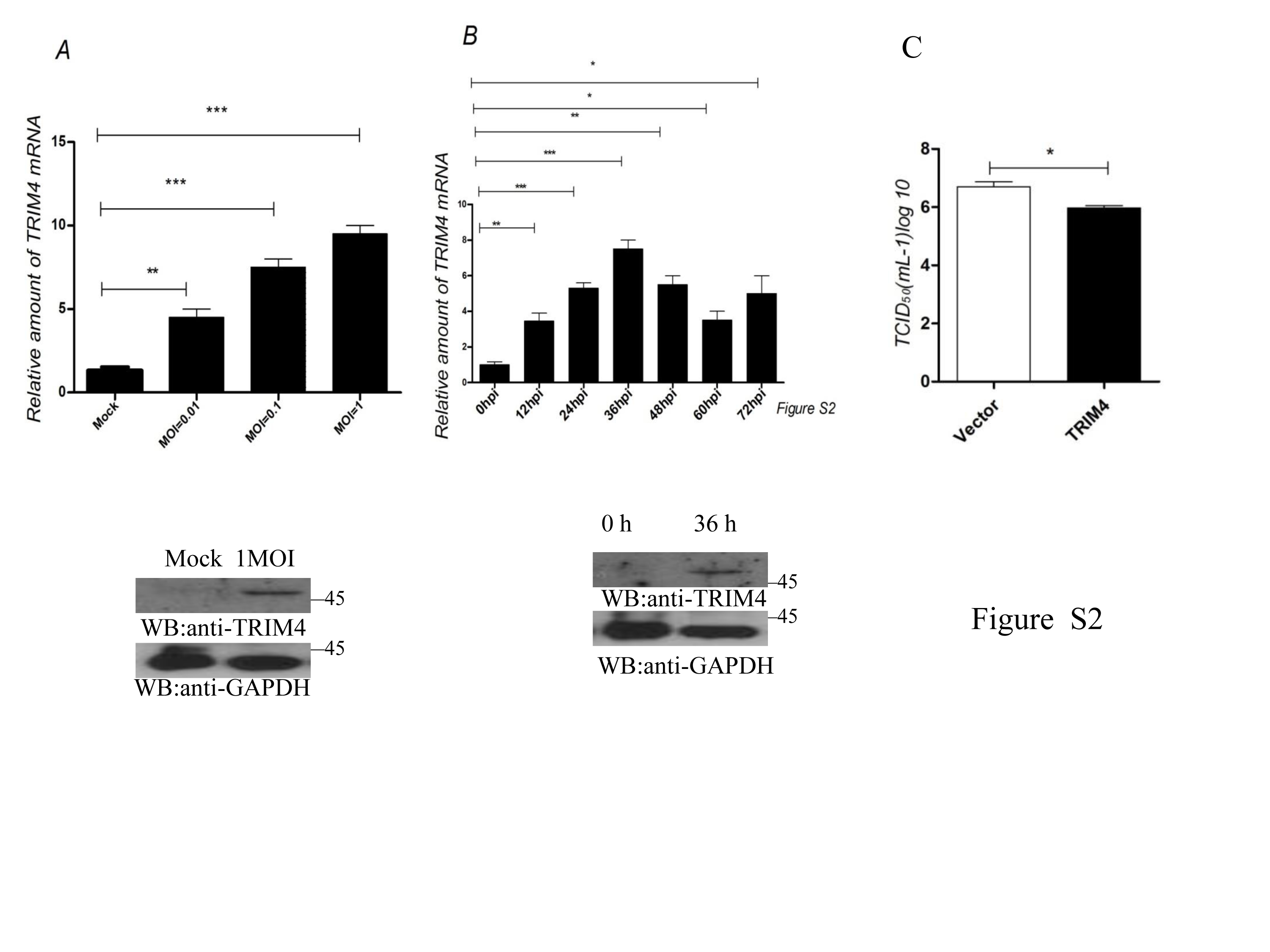

Supplement: Supplementary Figure 2 — Effects of PRRSV infection on TRIM4 expression and effects of TRIM4 on PRRSV replication. (A) Evaluation of different PRRSV MOI on TRIM4 expression. MARC145 cells were infected with different PRRSV MOIs (0.01, 0.1, and 1), and after 48 h, the changes in TRIM4 mRNA expression were measured by qPCR and western blotting. (B) Evaluation of different time points with the same PRRSV MOI on TRIM4 expression. MARC145 cells were infected with 1 MOI PRRSV, and changes in TRIM4 expression were examined by qPCR and western blotting at different time points (0, 12, 24, 36, 48, 60, and 72 h). (C) TRIM4 overexpression inhibits PRRSV replication. FLAG-TRIM4 (500 ng) was transfected into Marc-145 cells; cells were inoculated with 1 MOI PRRSV after 24 h, and the supernatant of cells was collected after 48 h post-infection, the virud titer was measured by TCID50. [file Image_2.jpg]
